# Supplementary material for: A Humanized Yeast Phenomic Model of Deoxycytidine Kinase to Predict Genetic Buffering of Nucleoside Analog Cytotoxicity
Source: Genes (Basel). 2019 Sep 30;10(10):770. doi: 10.3390/genes10100770 (PMC6826991; doi:10.3390/genes10100770)
Supplement: Supplementary file 1 [file genes-10-00770-s001.zip › Additional_Files/Additional_File7_GOTermHeatmaps.zip!/Additional_File7_GOTermHeatmaps/post-mRNA_release_spliceosomal_complex.pdf]

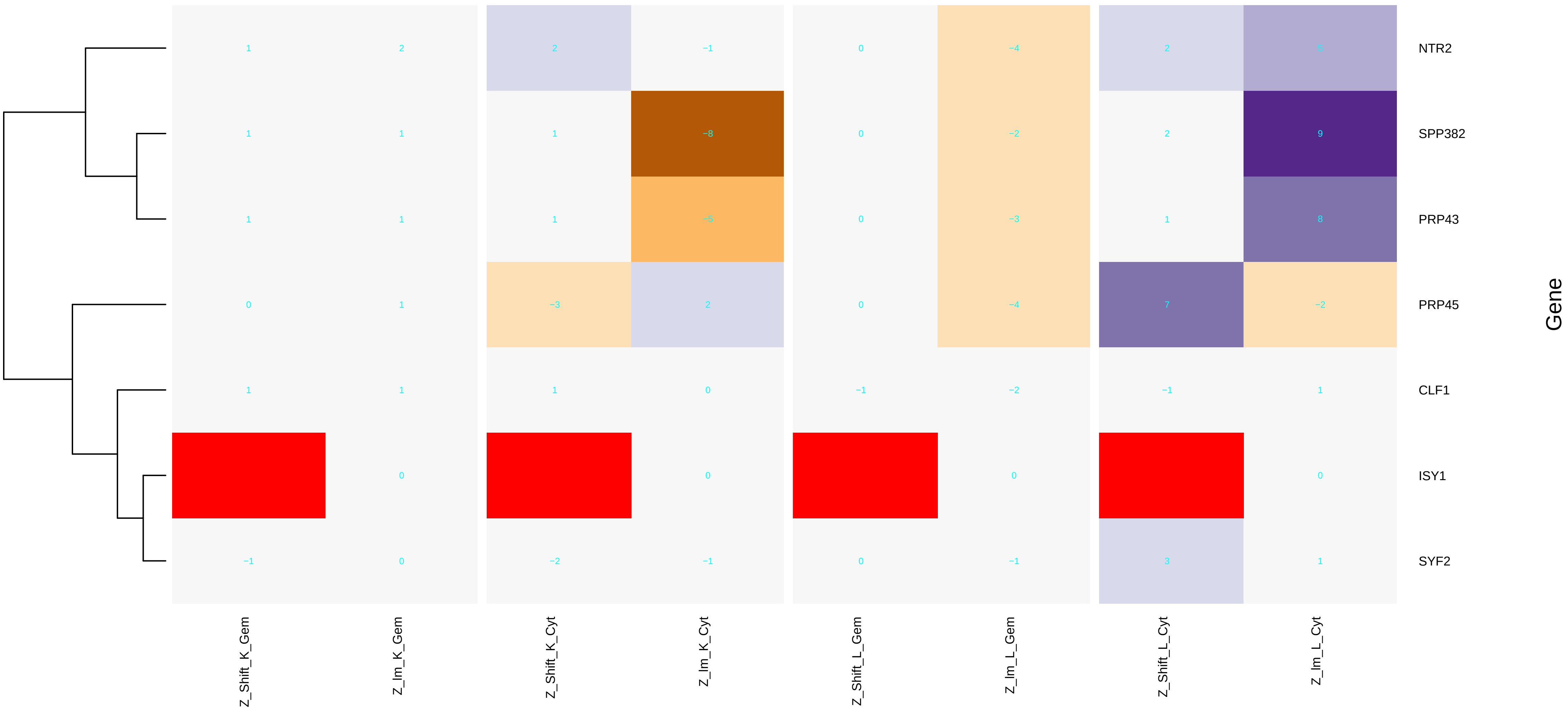

NTR2
SPP382
PRP43
PRP45
CLF1
ISY1
SYF2
Gene
Z_Shift_K_Gem
Z_Im_K_Gem
Z_Shift_K_Cyt
Z_Im_K_Cyt
Z_Shift_L_Gem
Z_Im_L_Gem
Z_Shift_L_Cyt
Z_Im_L_Cyt
1 2 2 −1 0 −4 2 5
1 1 1 −8 0 −2 2 9
1 1 1 −5 0 −3 1 8
0 1 −3 2 0 −4 7 −2
1 1 1 0 −1 −2 −1 1
0 0 0 0
−1 0 −2 −1 0 −1 3 1
